# Emerging Rickettsioses of the Thai-Myanmar Border^1^

**DOI:** 10.3201/eid0905.020511

**Published:** 2003-05

**Authors:** Philippe Parola, R. Scott Miller, Philip McDaniel, Sam R. Telford, Jean-Marc Rolain, Chansuda Wongsrichanalai, Didier Raoult

**Affiliations:** * Armed Forces Research Institute of Medical Sciences (AFRIMS), Bangkok, Thailand; † Unité de Médecine, Marseille, France; ‡ Harvard School of Public Health, Boston, Massachusetts, USA; § Kwai River Christian Hospital, Sangkhlaburi, Kanchanaburi, Thailand

**Keywords:** rickettsioses, *Rickettsia felis*, *Rickettsia typhi*, *Rickettsia conorii*, *Rickettsia helvetica*, *Orientia tsutsugamushi*, Thailand, dispatch

## Abstract

To investigate the presence of rickettsioses in rural residents of the central Thai-Myanmar border, we tested the blood of 46 patients with fever. Four patients had murine typhus, three patients had scrub typhus, and eight patients had spotted fever group rickettsioses, including the first case of *Rickettsia felis* infection reported in Asia.

Human rickettsioses known to occur in Thailand include mainly murine typhus and scrub typhus. Murine typhus is caused by *Rickettsia typhi* and is primarily maintained by fleas such as *Xenopsylla cheopis,* with various rodents reservoirs (*1*). Scrub typhus is caused by *Orientia tsutsugamushi* (formerly named *R. tsutsugamushi*), which is transmitted by the bites of the larvae of several species of trombiculid mites (commonly called “chiggers”) (*2*).

Spotted fever group (SFG) rickettsioses are associated with arthropods, mainly ticks but mites and fleas as well (*3*,*4*). In Thailand, few reports of serologically documented cases of SFG rickettsioses have been published (*5*). Although the specific etiologic agents of these diseases have not been identified, several SFG rickettsiae have been identified from ticks in Thailand. Thai tick typhus *Rickettsia* TT-118 was isolated from a pool of ticks in the 1970s (*6*). Despite its name, its pathogenic role in Thailand is not known. However, Stenos et al. have suggested that TT-118 is a strain of *R. honei,* an emerging pathogen prevalent on Flinders Island, Australia (*7*). Further, a rickettsia identified as *R. honei* type strain has also been recently detected by molecular methods in Thai *Ixodes granulatus* (*8*). In addition, several previously unrecognized rickettsiae of unknown pathogenicity have been detected from *Ixodes* and *Dermacentor* ticks, including species known to bite humans (*8*,*9*).

Thailand’s Sangkhlaburi District (Kanchanaburi Province) is a major gateway on the central part of the Thai-Myanmar border where newly arrived migrants from Myanmar become established as farm or factory laborers. There, the local Thai people, as well as Karen, Mon, and Burmese migrants, are commonly bitten by arthropods when working in the fields or at home. Scrub typhus has been previously reported in the province (*10*). Murine typhus was also described as a cause of fever in refugee camps along the Thai-Myanmar border (*11*). However, a serosurvey undertaken in 1997 suggested that residents of Sangkhlaburi were commonly exposed not only to the agents of scrub typhus and murine typhus but also to SFG rickettsiae and agents of human ehrlichioses (*12*). Here, we provide for the first time a more precise indication of rickettsioses in febrile patients from Sangkhlaburi.

## The Study

This study was based at the Armed Forces Research Institute of Medical Sciences (AFRIMS)–Kwai River Christian Hospital Clinical Center, Sangkhlaburi District, Kanchanaburi Province, Thailand. (The protocol was approved by the Human Subjects Research Review Board of the U.S. Army, Ethical Review Committee for Research in Human Subjects of the Thai Ministry of Public Health, and Scientific Review Committee of AFRIMS.) Patients were selected from those enrolled and sampled from June 1999 to February 2002 in an on-going “fever study,” which focuses on the etiology of undifferentiated febrile illnesses (oral temperature >38°C or history of fever within the past 48 h) in local residents >20 years of age. Criteria leading to the suspicion of rickettsioses included 1) a rash or eschar, 2) arthropod bites or recent exposure to the jungle, 3) a negative Giemsa-stained malaria smear, and 4) serum specimens that tested positive by enzyme-linked immunosorbent assay (ELISA) for SFG–specific immunoglobulin (Ig) M (PanBio, Brisbane, Australia) or dot-ELISA for total Ig of *R. rickettsii* or *R. typhi* (PanBio-INDX, Baltimore, MD). Serum specimens were sent to the Unité des Rickettsies in Marseille for specific diagnosis of rickettsioses. Serologic testing was performed by indirect immunofluorescence (IF) on acute-phase (day 0) and convalescent-phase (approximately day 21) samples. Serum specimens were tested by using a panel of 13 rickettsial antigens, including SFG rickettsiae (*R. conorii* Indian, *R. japonica*, *R. honei*, *R. helvetica*, *R. slovaca*, AT1 *Rickettsia* [*13*], *R. felis*, “*R. heilongjiangii”*) typhus group rickettsiae (*R. typhi*), *Orientia tsutsugamushi* (strain Gilliam, Kato, Karp, and Kawazaki), *Anaplasma phagocytophilum, Ehrlichia chaffeensis,* and *Coxiella burnetii*. The rationale for the antigen screening panel included the presence of the strains in Asia and results of previous serosurveys for *A. phagocytophilum* and *E. chaffeensis*. The standard procedure was followed for the use of Western blot and cross-adsorption studies to complete the IF assay at the Unité des Rickettsies (*14*,*15*). An immunofluorescence assay was considered positive for 1) IgG with titers >128 and/or IgM titers >64 for *R. conorii;* and 2) for IgG titers >64 and/or IgM titers >32 for other rickettsial antigens. When cross-reactions were noted between several rickettsial antigens, the standard procedure comprised three steps: 1) A rickettsial antigen was considered to represent the agent of infection when titers of IgG and/or IgM antibody against this antigen were at least two serial dilution higher than titers of IgG and/or IgM antibody against other rickettsial antigens. 2) When the difference in titers between several antigens was lower than two dilutions, Western blot assays were performed. A rickettsial antigen was considered to represent the agent of the infection when acute-phase or convalescent-phase sera reacted only with the specific proteins of this antigen. 3) When Western blot assays were not diagnostic, cross-absorption studies were performed: IgG/IgM titers had to be >128/32. Specific diagnosis criteria after cross-absorption studies included a) IF serologic test results positive for a single antigen or b) a Western blot assay showing an exclusive reactivity with specific proteins of a sole agent.

From June 1999 to February 2002, 46 patients were selected to be specifically tested for rickettsioses. These 46 patients were empirically treated by a 7-day doxycycline regimen (200 mg/d). Rickettsioses were serologically confirmed in 15 (33%) patients by evidence of seroconversion, IgM at significant titers, or both. Three patients (nos. 1–3) had scrub typhus caused by *O. tsutsugamushi*. Serum specimens from patients 1 and 3 provided the highest titers against Gilliam and Karp strains, and serum from patient 2 had titers against Gilliam strain only. No further study was conducted to identify the strain responsible for the disease. Two of these patients had returned from a trip into the jungle, and the third became sick several days after cutting grass in the fields. One patient was initially thought to have bacterial meningitis and had been treated unsuccessfully by a broad-spectrum third-generation cephalosporin for 3 days before doxycycline was started. Four patients (nos. 4–7) had murine typhus caused by *R. typhi*. All had fever and unspecific signs. The patients recalled no arthropod bite, and none had a rash. Eight cases were SFG rickettsioses (nos. 8–15). Of the patients with SFG rickettsioses, only one (no. 9) had fever, eschar, and rash. One patient (no.13) had an eschar at a tick bite site, and another had a rash (no.15). Others presented with unspecific signs. Cross-reactions were noted mostly within the SFG rickettsia antigens. One patient (no. 8) with SFG rickettsioses seroconverted to *R. felis*, indicated by high level of antibody titers. Further, although IgG titers were more than two serial dilutions higher than those for *R. typhi*, Western blot assay was performed to confirm IF findings. Two patients (nos. 9 and 10) were shown to have the highest titers to *R. conorii* strain Indian. Five patients (nos. 11–15) had the highest titers to *R. helvetica*. For patients 4, 9, 10, 13, and 15, IF results showed differences lower than two dilutions in IgG titers, IgM titers, or both, between several antigens. Thus, IF assays were completed by Western blot and with cross-absorption studies for patient 4 (Table). No cases of infection due to *C. burnetii* or ehrlichioses were diagnosed in the 46 tested patients.

**Table:** Clinical and laboratory data of patients with rickettsioses on the Thai-Myanmar border.

Patient no.	Age/sex	A. bite	Clinical signs accompanying fever				Immunofluorescence serologic testing^a^ IgG/IgM early IgG/IgM late												
			R	E	N	Other	SFG rickettsia antigens								*R. typhi* antigen	**Orientia tsutsugamushi**			
							Rh	Rc	Rjap	Rhon	Rslo	AT1	Rheil	Rfel		G	Kw	Kp	K	
Scrub typhus																				
1	29/M	Not noticed	No	No	Yes	Headache, stupor, meningism, thrombocytopenia, ↑ALT	0/16 0/16	0/0 0/0	0/16 0/16	0/8 0/8	0/0 0/0	0/0 0/0	0/0 0/0	0/0 0/0	0/0 0/0	0/32 256/32	0/8 0/8	0/8 256/32	0/0 0/0	
2	50/F	Yes	Yes^b^	Yes	No	Chills, myalgia, vomiting, thrombocytopenia, ↑GGT ↑ALT	0/32 0/32	0/0 0/0	0/32 0/32	0/16 0/16	0/0 0/0	0/0 0/0	0/0 0/0	0/0 0/0	0/0 0/0	128/64 128/16	0/0 0/0	0/0 0/0	0/0 0/0	
3	32/M	Yes	No	No	Yes	Headache, myalgia, cough, thrombocytopenia, ↑GGT ↑ALT	0/16 16/16	0/0 0/0	0/0 16/16	0/8 0/8	0/0 0/0	0/0 0/0	0/0 0/0	0/0 0/0	0/0 0/0	0/0 512/128	0/0 0/0	0/0 512/0	0/0 0/0	
Murine typhus																				
**4**	28/F		No	No	No	Chills, headache, vomiting, myalgia, cough, thrombocytopenia	16/128^e^ 16/64^e^	16/64 16/64	16/8 0/0	16/16 16/64	16/16 16/8	0/0 0/0	0/32 0/8	0/0 0/0	**64/128**^e^ **32/32^e^**	0/0 0/0	0/0 0/0	0/0 0/0	0/0 0/0	
**5**	35/M	Not noticed	No	No	No	Chills, headache, ↑ALT	0/64 16/128	0/64 16/256	0/8 0/8	0/64 0/64	0/0 0/0	0/0 0/0	0/32 0/128	0/0 0/0	**16/256** **512/256**	0/0 0/0	0/0 0/0	0/0 0/0	0/0 0/0	
6	37/F	Not noticed	No	No	No	Chills, headache, myalgia, vomiting, thrombocytopenia, ↑GGT ↑ALT	16/64 128/64	32/8 32/0	0/0 32/0	16/0 128/0	16/0 128/8	0/0 32/8	0/0 32/0	0/0 0/0	**64/8** **512/256**	0/0 0/0	0/0 0/0	0/0 0/0	0/0 0/0	
7	20/F	Not noticed	No	No	No	Headache, back pain	0/128 16/64	0/64 0/64	0/0 0/64	0/32 0/64	0/0 0/164	0/0 0/8	0/0 0/0	0/0 0/0	**0/64** **128/256**	0/0 0/0	0/0 0/0	0/0 0/0	0/0 0/0	
SFG rickettsioses																				
8	70/F		No	No	No	Chills, headache, vomiting, hepatomegaly, leukopenia	0/32 32/32	0/32 32/32	0/0 0/0	0/8 16/8	0/0 0/0	0/0 0/0	0/0 0/0	**0/0** **1024/256**^d^	0/0 0/256^d^	0/0 0/0	0/0 0/0	0/0 0/0	0/0 0/0	
9	50/M	Not noticed	Yes^c^	Yes	Yes	Chills, abdominal pain, confusion, thrombocytopenia	64/8^d^ 64/16	**128/16**^d^ **128/16**	32/0^d^ 32/0	32/0^d^ 64/0	32/0^d^ 128/0	0/0 0/0	16/0**^d^** 32/8	0/0 0/0	0/0 0/0	0/0 0/0	0/0 0/0	0/0 0/0	0/0 0/0	
10	45/M		No	No	Yes	Vomiting, diarrhea, hepato-splenomegaly	16/32^d^ 64/32	**64/32**^d^ **64/32**	16/32^d^ 32/16	16/0^d^ 32/16	16/16^d^ 16/16	0/0 0/0	16/8^d^ 32/16	0/0 0/0	0/16^d^ 16/32	0/0 0/0	0/0 0/0	0/0 0/0	0/0 0/0	
11	35/M	Tick bite	No	Yes	No	Chills, headache, vomiting, myalgia, cough, splenomegaly, ↑GGT ↑ALT	**64/256** **16/128**	0/16 16/32	16/8 0/8	16/256 16/128	0/64 0/64	0/32 16/8	32/32 0/16	0/0 0/0	0/256 0/256	0/0 0/0	0/0 0/0	0/0 0/0	0/0 0/0	
12	37/F	A. removed from ear	No	No	No	Chills, headache, vomiting, myalgia, cough, ↑GGT ↑ALT	**0/16** **128/32**	0/0 0/0	0/0 64/0	0/0 32/16	0/0 16/32	0/8 0/0	0/8 32/16	0/0 0/0	0/32 0/64	0/0 0/0	0/0 0/0	0/0 0/0	0/0 0/0	
13	20/F	Tick bite	No	Yes	No	Headache, chills, myalgia	**32/16**^d^ **64/16**	32/0^d^ 32/0	32/0^d^ 64/0	64/0^d^ 128/0	32/8^d^ 64/8	0/0 0/0	0/0 0/0	32/0^d^ 64/0	0/0	512/0 512/0	0/0	0/0	0/0	
14	55/F		No	No	No	Chills, headache, myalgia, diarrhea, thrombocytopenia	**16/32** **128/16**	16/16 16/16	16/16 16/16	16/16 16/16	16/16 64/0	16/16 16/16	16/0 64/0	32/0 64/0	0/0 0/0	0/0 0/0	0/0 0/0	0/0 0/0	0/0 0/0	
15	29/M		Yes^b^	No	No	Chills, headache, myalgia, cough, thrombocytopenia, ↑GGT ↑ALT	**16/16** **64/64**^d^	16/16 32/32^d^	32/16 32/32**^d^**	32/16 32/32^d^	16/0 64/64^d^	32/16 32/32^d^	16/0 64/64^d^	16/16 64/64^d^	0/0 0/0	256/0 256/0	256/0 256/0	0/0 0/0	0/0 0/0	

Abbreviations: A., arthropod; Ig, immunoglobulin; R, rash; E, eschar; N, nodes; Rh, *Rickettsia helvetica*; Rc, *R. conorii* Indian; Rjap, *R. japonica*; Rhon, *R. honei*; Rslo, *R. slovaca*; AT1, *Rickettsia* strain AT1 from Japan; Rheil, “*R. heilongjiangii”*; Rfel, *R. felis*; G, strain Gilliam; Kw, strain Kawazaki; Kp, strain Karp; Ko, strain Kato. ^a^Immunofluorescence assay was completed by Western blot and cross-adsorption as described in the text. For typhus and spotted fever group antigens, the rickettsia considered potentially responsible for the infection are in bold type. ^b^Maculopapular over chest and back. ^c^Vesiculous on the legs. ^d^Antigens and sera used for Western blot assays. ^e^Antigens and sera used for Western blot and cross-adsorption assays.

## Conclusions

In this study, we report rickettsioses in Sangkhlaburi, including the first case of *R. felis* infection reported in Asia. *R. felis* is an emerging pathogen responsible for flea-borne spotted fever. *R. felis* was likely first detected (as *R. ctenocephali*) in European cat fleas (*Ctenocephalides felis)* in 1918 (*16*), then rediscovered in 1990 in the United States (*17*). *R. felis* was then cultivated and characterized as a unique SFG rickettsia (*18*). Its pathogenic role was recently demonstrated in patients with serologic evidence of infection in Brazil, France, and Germany. *R. felis* DNA has also been detected in sera in Texas, Mexico, Brazil, and Germany (*19*). This rickettsia has also been recently detected in fleas in Brazil, Africa, Spain, and France (*20*). Further, during an entomologic survey, *R. felis*–like rickettsiae were detected in fleas collected in Sanghklaburi (P. Parola, unpub. data). These data suggest that *R. felis* infection is endemic in Sanghklaburi and perhaps globally.

Murine typhus, a mild disease with nonspecific signs (*21*), was found in four of our patients. Although this disease has a worldwide distribution, it is often unrecognised, and documented cases are rarely reported. The classic triad of fever, headache, and skin rash is observed in <15% of cases (*22*). For example, our four patients did not have a rash. Arthralgia, myalgia, and respiratory and gastrointestinal symptoms (as demonstrated by one of our patients) are frequent (*21*,*22*). Regarding disease transmission, although rats and mice are very common within and around houses in the villages, our patients did not report contact with rat fleas or a flea bite.

In this study, seven patients with SFG rickettsioses may have been infected by *R. helvetica* (five patients) or *R. conorii* Indian strain (two patients), according to IF assays completed for some cases by Western blot and cross-adsorption studies. *R. helvetica* is an emerging pathogen known to be prevalent in Europe (*23*) and Japan (*13*). In both areas, *R. helvetica* is associated with *Ixodes* ticks, which are also found in Thailand, although they have not previously been reported in Sangkhlaburi (*24*). *R. conorii* Indian is known as an agent of tick-borne rickettsioses prevalent in India, where it is associated with the dog tick (*Rhipicephalus sanguineus)* (*25*), which is found worldwide. However, an unknown *Rickettsia* sp. that is cross-reactive with *R. conorii* Indian and *R. helvetica* could also be responsible for the cases reported here. In particular, we have recently detected, by polymerase chain reaction, *Rickettsia* spp. from ticks that have bitten people in the Sangkhlaburi area, including *Dermacentor auratus* and *Dermacentor* sp*.* larvae (*9*). The pathogenic role of these rickettsiae has yet to be demonstrated.

Scrub typhus is essentially an occupational disease among rural residents in the Asia-Pacific region (*2*). This disease is often underdiagnosed or misdiagnosed when the classic eschar at the chigger bite sites and the rash are absent, as reported for two of our three patients (*2*). The severity of the disease varies from asymptomatic to fatal (up to 30%). Delayed or inappropriate treatment such as with third-generation cephalosporins, as reported for one of our patients, is associated with a severe outcome. The four major serotypes studied here have been shown to have sufficient cross-reactivity with antigens from other strains to be used for serologic diagnostic testing. In our patients, although the highest titers were obtained by using *O. tsutsugamushi* strain Gilliam antigens, other strains that share common epitopes and cross-react with this strain could be involved.

Patients with rickettsioses may have isolated fever or fever with nonspecific clinical and laboratory findings. These diseases are easily misdiagnosed because rash or eschar (the hallmark for rickettsial diseases) is absent, the diseases are not recognized by local physicians, or the diseases have never been reported in the area. More studies are needed on tropical rickettsioses, in particular, molecular detection or rickettsial isolation from patient samples, complemented by detailed case reports. Studying possible vectors and animal reservoirs would provide estimates of the degree of zoonotic potential. Ultimately, such studies will provide the basis for determining prevalence of rickettsiosis in the tropics and their effects on public health.
